# Extracellular Vesicles in Serum and Central Nervous System Tissues Contain microRNA Signatures in Sporadic Amyotrophic Lateral Sclerosis

**DOI:** 10.3389/fnmol.2021.739016

**Published:** 2021-10-29

**Authors:** Ting-wen Lo, Claudia Figueroa-Romero, Junguk Hur, Crystal Pacut, Evan Stoll, Calvin Spring, Rose Lewis, Athul Nair, Stephen A. Goutman, Stacey A. Sakowski, Sunitha Nagrath, Eva L. Feldman

**Affiliations:** ^1^Department of Chemical Engineering, University of Michigan, Ann Arbor, MI, United States; ^2^Department of Neurology, University of Michigan, Ann Arbor, MI, United States; ^3^Department of Biomedical Sciences, School of Medicine and Health Sciences, University of North Dakota, Grand Forks, ND, United States; ^4^Binterface Institute, University of Michigan, Ann Arbor, MI, United States; ^5^Rogel Cancer Center, University of Michigan, Ann Arbor, MI, United States

**Keywords:** amyotrophic lateral sclerosis, biomarker, central nervous system, extracellular vesicle, microRNA, neurodegeneration, pathway analysis, serum

## Abstract

Amyotrophic lateral sclerosis (ALS) is a terminalneurodegenerative disease. Clinical and molecular observations suggest that ALS pathology originates at a single site and spreads in an organized and prion-like manner, possibly driven by extracellular vesicles. Extracellular vesicles (EVs) transfer cargo molecules associated with ALS pathogenesis, such as misfolded and aggregated proteins and dysregulated microRNAs (miRNAs). However, it is poorly understood whether altered levels of circulating extracellular vesicles or their cargo components reflect pathological signatures of the disease. In this study, we used immuno-affinity-based microfluidic technology, electron microscopy, and NanoString miRNA profiling to isolate and characterize extracellular vesicles and their miRNA cargo from frontal cortex, spinal cord, and serum of sporadic ALS (*n* = 15) and healthy control (*n* = 16) participants. We found larger extracellular vesicles in ALS spinal cord versus controls and smaller sized vesicles in ALS serum. However, there were no changes in the number of extracellular vesicles between cases and controls across any tissues. Characterization of extracellular vesicle-derived miRNA cargo in ALS compared to controls identified significantly altered miRNA levels in all tissues; miRNAs were reduced in ALS frontal cortex and spinal cord and increased in serum. Two miRNAs were dysregulated in all three tissues: miR-342-3p was increased in ALS, and miR-1254 was reduced in ALS. Additional miRNAs overlapping across two tissues included miR-587, miR-298, miR-4443, and miR-450a-2-3p. Predicted targets and pathways associated with the dysregulated miRNAs across the ALS tissues were associated with common biological pathways altered in neurodegeneration, including axon guidance and long-term potentiation. A predicted target of one identified miRNA (N-deacetylase and N-sulfotransferase 4; NDST4) was likewise dysregulated in an *in vitro* model of ALS, verifying potential biological relevance. Together, these findings demonstrate that circulating extracellular vesicle miRNA cargo mirror those of the central nervous system disease state in ALS, and thereby offer insight into possible pathogenic factors and diagnostic opportunities.

## Introduction

Amyotrophic lateral sclerosis (ALS) is a heterogeneous fatal neurodegenerative disorder. Neurodegeneration and voluntary skeletal muscle atrophy proceed in a progressive manner, resulting in respiratory failure and death within 3–5 years of symptom onset. Most cases are sporadic, with only 15% of cases caused by known genetic mutations. Riluzole, edaravone, and comprehensive patient care are the only available interventions for ALS and only marginally improve patient survival (Gittings and Sattler, 2020). The unknown disease etiology and lack of sensitive diagnostic and prognostic biomarkers present a challenge for drug development.

Extracellular vesicles (EVs) are ubiquitously secreted membrane-encapsulated vesicles and are stable in biological fluids and tissues (Boukouris and Mathivanan, 2015). EVs contain molecular cargo from cells, such as genetic material, proteins, metabolites, and cellular waste, and travel via the circulation to recipient cells to execute specific cellular functions (Margolis and Sadovsky, 2019). Changes in EV levels and cargo content are associated with health and disease, as well as responses to extracellular stimuli (Datta Chaudhuri et al., 2019; Margolis and Sadovsky, 2019). In the nervous system, EVs may promote neurodegeneration by transferring pathogenic molecules between neuronal and non-neuronal cells in a prion-like manner, which can “spread” disease (Soria et al., 2017; McAlary et al., 2020). Importantly, EVs also cross the blood-brain barrier (Saint-Pol et al., 2020), making them an attractive biomarker candidate for diseases of the central nervous system (CNS), including ALS. Indeed, EV cargo in cellular ALS models contain misfolded and aggregated proteins, as well as dysregulated microRNAs (miRNAs) and mRNAs (Ferrara et al., 2018). However, EV mobility, cargo, molecular mechanisms of action, and diagnostic implications are still not well understood in ALS, and continued research is needed (Gagliardi et al., 2021).

High purity EVs can be quickly and selectively isolated from small amounts of bio-samples utilizing microfluidic immuno-affinity technology paired with specific antibodies that recognize EV surface antigens. In this study, we optimized the ExoChip (Kanwar et al., 2014), an immuno-affinity-based microfluidic device, to isolate and quantitate EVs from sporadic ALS and control frozen postmortem CNS tissue and serum. We then characterized the EV cargo, specifically focusing on miRNAs. These small non-coding RNAs regulate gene expression post-transcriptionally by destabilizing multiple target mRNAs and thereby modulate physiological and pathological processes. Importantly, the presence of dysregulated miRNAs in ALS biofluids and postmortem tissue across multiple studies supports the contention that miRNAs exert important functions in disease pathology (Paez-Colasante et al., 2015; Wang and Zhang, 2020; Akbari Dilmaghani et al., 2021). Results from the current study provide a foundation for leveraging miRNA cargo from circulating EVs as a reflection of pathological EV signatures in ALS CNS tissue, generating new insight into possible pathogenic factors and untapped diagnostic opportunities.

## Materials and Methods

### Study Participants and Sample Processing

Sporadic non-familial ALS participants were recruited at the Pranger ALS Clinic at Michigan Medicine. Disease status was determined by an ALS neurologist based on revised El Escorial World Federation of Neurology criteria (Brooks et al., 2000), electromyography, and clinical and family history data. Age- and sex-matched control participants were recruited via a university-managed recruitment website and were eligible to participate if they: (1) did not have an ALS diagnosis or other neurodegenerative condition, and (2) did not have a family history of ALS in a first- or second-degree blood relative. Adult participants and/or next of kin provided informed consent. This research was approved by the University of Michigan Medical School Institutional Review Board (HUM00028826).

Whole blood samples from participants were drawn and collected in EDTA tubes, and serum was obtained as previously reported (Kanwar et al., 2014). Postmortem CNS tissue homogenates were obtained from approximately 100 mg of frozen frontal cortex and 300 mg of spinal cord as previously reported (Banigan et al., 2013) for participants with available tissue. Tissue homogenates were filtered through a 0.2 μm syringe filter (MilliporeSigma, Burlington, MA, United States). A 1:10 dilution was used to determine protein concentration using a BCA kit (Pierce Biotechnology, Waltham, MA, United States).

### ExoChip Device Fabrication and Functionalization

The ExoChip device was fabricated as previously reported (Kanwar et al., 2014). Briefly, a polydimethylsiloxane layer was bonded to glass substrate to form a microfluidic chamber with a height of 50 μm. The device was then chemically functionalized with NeutrAvidin (Thermo Fisher Scientific, Waltham, MA, United States) and stored at 4 °C until use. Biotinylated anti-CD63 (Ancell, Bayport, MN, United States) was added before EV isolation to target tetraspanin protein CD63, a common EV marker (Jankovicova et al., 2020).

### Extracellular Vesicle On-Chip Capture

EVs were isolated by immuno-affinity as previously reported (Kanwar et al., 2014). The saturation capacity of the ExoChip device was first determined using 300 μL of tissue homogenates with total protein concentrations ranging from 0.25 to 1 μg/μL for frontal cortex and 0.5–1.167 μg/μL for spinal cord, or volumes ranging from 200 to 450 μL for serum. This provided the guiding parameters for EV capture to assess EV number, which used sub-saturated conditions to avoid device overload, or to assess EV size, which used saturated conditions to ensure maximal EV capture. Frontal cortex or spinal cord homogenates or serum were infused through the ExoChip at a flow rate of 6 μL/min, followed by a rinse with tris buffered saline at a flow rate of 50 μL/min for 20 min. To measure the number of EVs, 300–350 μL of tissue homogenates with protein concentrations adjusted to 0.3 μg/μL, or 300–350 μL of serum, were processed through the device. To harvest the EV cargo, 300–350 μL of tissue homogenates with protein concentrations adjusted to 1 μg/μL, or 1 mL of serum, was used. The immobilized EVs were stained with membrane dye for on-chip quantification, fixed for scanning electron microscopy (SEM), or lysed for protein and miRNA extraction (see relevant sections below).

### Extracellular Vesicle Membrane Labeling and Quantification

To quantify EV number, captured EV membrane-associated lipids were stained by flushing the chambers with the fluorescent carbocyanine dye, Vybrant^TM^ DiO (Molecular Probes, Eugene, OR, United States) at a flowrate of 20 μL/min for 10 min, followed by incubation with the DiO dye at 37°C for 20 min. Fluorescence intensities were measured at an excitation wavelength of 485 nm and an emission wavelength of 510 nm using a BioTek Synergy Neo multi-purpose plate reader (Winooski, VT, United States). Fluorescence intensities were normalized to the background.

### Preparation for Scanning Electron Microscopy and Vesicle Size and Morphology Analysis

To prepare captured EVs for SEM, standard SEM preparation (Kanwar et al., 2014) was performed with the following modifications. ExoChip immobilized EVs were fixed with 2% paraformaldehyde in phosphate-buffered saline (PBS) for 1 h at room temperature and rinsed with PBS. Next, the samples were serially dehydrated for 10 min in ethanol (30%, 50%, 70%, 95% in distilled water, and 100%) followed by a 10 min incubation in 1:1 ethanol:hexamethyldisilazane (HMDS), transferred to 100% HMDS, and air dried overnight. The samples were then gold- or carbon-coated for SEM imaging at 25,000× or 35,000× magnification with a FEI Nova 200 NanoLab Dual-Beam FIB-SEM microscope under low beam energies (2.0–5.0 kV) at the Michigan Center for Materials Characterization at the University of Michigan.

EV size and morphology were analyzed in SEM micrographs from 3 ALS and 3 control participants using MetaMorph image analysis software (version 7.7.7.0, Molecular Devices, San Jose, CA, United States). Briefly, the threshold feature was used to identify EVs. After all objects were isolated, EVs were verified using a filter for total EV area and shape factor (0 indicated a straight line and 1 indicated a perfect circle). Objects smaller than 2000 nm^2^ and with a shape factor lower than 0.2 were discarded from the data set (Kanwar et al., 2014).

### Protein Analysis by Western Blotting

To lyse the captured EVs, the devices were flushed with RIPA buffer (Sigma, St. Louis, MO, United States) supplemented with cOmplete, EDTA-free protease inhibitor tablets (Roche, Basel, Switzerland) at a flowrate of 20 μL/min. The resulting protein lysates were analyzed by standard Western blotting procedures using 4–20% gradient acrylamide gels (Bio-Rad Laboratories, Hercules, CA, United States) and transferred to nitrocellulose membranes (Amersham, St. Louis, MO, United States) per our standard protocol (Kanwar et al., 2014). Immunoblotting was performed using rabbit anti-CD9 [Cell Signaling Technologies (CST), Danvers, MA, United States, cat# 13174] or anti-β-actin (CST, cat#4970) primary antibodies, followed by horseradish peroxidase-linked anti-rabbit IgG secondary antibody (CST, cat#7074). Proteins were visualized using Clarity ECL Western Blotting Substrates (Bio-Rad Laboratories) and a Bio-Rad ChemiDoc imager (Bio-Rad Laboratories).

### RNA Extraction and NanoString miRNAs Analysis

ExoChip captured EVs were lysed and flushed with Qiazol (QIAGEN, Hilden, Germany) at a flowrate of 20 μL/min. Total RNA was extracted following a standard phenol/chloroform protocol and Norgen columns (Norgen Biotek Corp, Thorold, ON, Canada) as indicated by the manufacturer. The samples were eluted in 50 μL of DNAse- and RNAse-free water and stored at –80°C.

EV miRNAs were profiled with the NanoString nCounter^®^ Human v3 miRNA Expression Assay Kit (NanoString, Seattle, WA, United States) at the Genomics Shared Resource at the Ohio State University Comprehensive Cancer Center according to the manufacturer and as previously reported (Tank et al., 2018). The nSolver (Wohlfarth et al., 2017) analysis platform was used to assess the quality of the nCounter data and process the data for further analysis. Normalization was done using NanoString’s three ligation control probes. Bioconductor package *sva* was used to remove batch effects from combining five different assay kits (Leek et al., 2012). Principal component analysis was used to visualize variation in differentially expressed miRNAs (DEmiRNAs) across samples. Probes were excluded from further analysis if their average count across all samples fell below the geometric mean plus one standard deviation (geomean + 1SD) of all negative controls. Differential miRNA expression between control and ALS groups in each tissue was evaluated using a two-tailed *t*-test, as used in NanoString nSolver v4.0 analysis software. The significance cutoff for DEmiRNAs was a nominal *P*-value < 0.05.

### Pathway Analysis

DEmiRNAs identified in the geomean + 1SD analysis were entered into the pathway analysis. Briefly, DIANA-miRPath v3.0 (Vlachos et al., 2015) was employed to collect predicted gene targets of all significant DEmiRNAs and to characterize associated biological functions and pathways, focusing on the Kyoto Encyclopedia of Genes and Genomes (KEGG) pathways. Kyoto Encyclopedia of Genes and Genomes pathways with a false discovery rate (FDR) < 0.05 were considered significantly enriched among the DEmiRNAs from each tissue type (frontal cortex, spinal cord, and serum). To identify the overall theme of these enriched pathways, an association network for each tissue was generated based on the gene-content overlap among the KEGG pathways using *richR*, our in-house analysis R package,^1^ and visualized in Cytoscape (McGregor et al., 2018). *P*-values were transformed by –log_10_(*P*-value) and color-indexed to indicate significance levels; significance ranged from no significance (white) to highest significance (dark red). The inter-relationship among the significant pathways across three tissues was analyzed in Cytoscape, and highly interconnected pathway clusters were detected by MCODE (Bader and Hogue, 2003), a Cytoscape app for network cluster analysis.

### Target Gene Expression Analysis in an *in vitro* Amyotrophic Lateral Sclerosis Model

Induced pluripotent stem cell (iPSC)-derived neurons (iNeurons) were established from control or sporadic ALS iPSC lines, as previously reported (Zhang et al., 2013; Tank et al., 2018; Weskamp et al., 2020). Briefly, iPSCs were plated on poly-D-lysine (50 μg/L, Sigma, cat# p1149)/laminin (1:100, Sigma, cat# L2020)-coated 6- well plates in iPSC media [E8 media (Gibco, cat# A1517001) supplemented with iROCK Y27632 (Fisher, cat# BDB562822)] at a density of 1 × 10^5^ cells/well. The following day (Day 1), the media was changed to iNeuron media #1 [E8 media supplemented with 1X N2 supplement (Gibco, cat# 17502-048), 1X NEAA supplement (Gibco, cat# 11140-050), 10 ng/mL BDNF (Peprotech, cat# 450-02), 10 ng/mL NT3 (Peprotech, cat# 450-03), 0.2 μg/mL mouse laminin (Sigma, cat# L2020), and 2 mg/mL doxycycline (Sigma, cat# D3447).] On Day 2, the culture media was changed to iNeuron media #2 [1/2 E8, 1/2 DMEM/F12 (Gibco, cat# 11320-033), 1X N2 supplement, 1x NEAA supplement, 10 ng/mL BDNF, 10 ng/mL NT3, 0.2 μg/mL laminin, 2 mg/mL Dox.] On Day 3, the culture media was changed to iNeuron media #3 [Neurobasal-A (Gibco, cat# 12349-015), 1x B27 supplement (Gibco, cat# 17504-044), 1x Glutamax supplement (Gibco, cat# 35050-061), 10 ng/mL BDNF, 10 ng/mL NT3, 0.2 μg/mL mouse laminin, 2 mg/mL doxycycline]. Additional media #3 was added on Day 6 and Day 8. The cells were differentiated for 10 days and maintained at 37°C and 5% CO_2_.

Next, EVs were isolated from ALS participant spinal cord tissue by ultracentrifugation. Briefly, ALS spinal cord (200 mg) was homogenized in 2 mL cold PBS supplemented with cOmplete mini, EDTA-free protease inhibitors (Roche, cat# 11836170001). The homogenate was passed 10 times through an 18-gauge needle and then repeated with a 20-gauge needle. The sample was centrifuged at 10,000 *g* at 4°C for 30 min. The supernatant was passed through a 0.22 μm filter (Millipore, Carrigtowhill, Cork, Ireland) and centrifuged at 100,000 *g* at 4°C in an Optima L-90K Ultracentrifuge (Beckman Coulter) for 3 h. The pelleted EVs were resuspended in 10 mL of 1X PBS and centrifuged at 100,000 *g* for 3 h at 4°C. EVs were resuspended in 500 μL 1X PBS with protease inhibitors. EV protein concentration was determined by BCA assay (Pierce Biotechnologies) and nanoparticle tracking analysis using a NanoSight NS300 (Malvern, United Kingdom).

Isolated EVs were incubated with control iNeurons for 8 h by replacing iNeuron media #3 with ∼5.3 × 10^8^ centrifugation extracted spinal cord EV particles resuspended in iNeuron media #3. Cells were then washed with 1X PBS and lysed in QIAzol (Qiagen, cat# 79306) prior to total RNA extraction by phenol/chloroform. The upper layer was passed through a miRNeasy Mini Kit column (Qiagen) and DNase treated using an RNAse-Free DNase Set (Qiagen, cat#79254). cDNA was generated using 1 μg of total RNA and the iScript cDNA synthesis kit (BioRad, cat# 1708840). Quantitative real-time PCR was performed in triplicate using TaqMan sequence specific probes for human N-deacetylase and N-sulfotransferase 4 (NDST4) and YWHAZ, TaqMan^TM^ 2X gene expression Master Mix (Applied Biosystems/Thermo Fisher Scientific, cat#4369016), and 2 μL of iNeuron cDNA in an Applied Biosystems StepOne^TM^ RT-PCR system thermocycler. NDST4 was selected by cross-referencing our EV DEmiRNA predicted targets with gene expression data from a previous analysis of ALS spinal cord (Figueroa-Romero et al., 2012). C_T_ values were used to calculate ΔC_T_ and ΔΔC_T_ using YHWAZ as the internal reference. Data were expressed as the mean of the relative quantity of gene expression (2^−ΔΔCT^).

### Statistical Analyses

Fisher’s exact test and Student’s *t*-test were used to determine significant demographic differences between ALS cases and controls. Three linear mixed effects models with random participant intercepts were used to determine differences in EV area between ALS participants and controls in the frontal cortex, spinal cord, and serum. The mixed effects models with random participant-specific intercepts identifies differences in EV area between ALS participants and controls, while accounting for the correlation of repeated EV area measurements within participants. The mixed effects models were fit using the lmerTest package in R software (v.3.5.2^2^) and model parameter estimates were determined using the maximum likelihood method (Kuznetsova et al., 2017). *T*-tests calculated using Satterthwaite’s degrees of freedom method were evaluated to assess differences in EV size between ALS participants and controls in the three regions (Giesbrecht and Burns, 1985). Statistically significant fold-change differences in gene expression between ALS and control iNeurons were calculated using a Student’s *t*-test. Statistical analyses were done using Prism (version 7 or 9.1.0; GraphPad Software, La Jolla, CA, United States) and R statistical computing software [v4.0.3 (see text footnote 2)]. Cumulative plots were generated using R software (v.4.0.1). Significance was defined as *P*-value < 0.05, unless noted otherwise.

## Results

### Participants and Samples

A total of 15 ALS and 16 control participants provided frontal cortex, spinal cord, and/or serum (Table 1 and Supplementary Table 1). All three tissues were collected from 11 of the ALS participants. None of the ALS participants exhibited any known familial history, *Cu^2+^/Zn^2+^ superoxide dismutase* (*SOD1*) gene mutations, or pathogenic *C9orf72* gene expansions. Age distribution and sex were similar between ALS and control participants. Fourteen ALS participants exhibited limb onset disease (93%), while one participant presented with bulbar onset (7%). All ALS participants were Caucasian (100%), while the control group consisted of 15 Caucasians (94%) and one Black/African American (6%).

**TABLE 1:** Clinical characteristics of study participants.

Characteristics		ALS group (*n* = 15)	Control group (*n* = 16)	*P*-value
Age (years)^(a,b,c)^		71.0, 64.4 ± 2.649, 47–80	60, 66 ± 2.954, (49–85)	0.726^#^
Sex	Male	8 (53.33%)	9 (56.25%)	>0.999^¶^
	Female	7 (46.67%)	7 (43.75%)	
Onset	Bulbar	1 (6.67%)	–	
	Limb	14 (93.33%)	–	
PMI ^(b)^*		13.73 ± 2.11 h (*n* = 11)	16.33 ± 2.08 h (*n* = 9)	0.396^#^
ALS Classification	Familial	0	–	
	Sporadic	16 (100%)	–	
Ethnicity	Caucasian	15 (100%)	15 (93.75)	
	Black/AA	–	1 (6.25%)	

(a) median; (b) mean ± standard error; (c) range; *For autopsy tissue, no data available from one participant. AA, African American; PMI, Postmortem Interval; ^#^student t-test; ^¶^Fisher’s exact test.

### Characterization of ExoChip Captured Extracellular Vesicles

**FIGURE 1:**
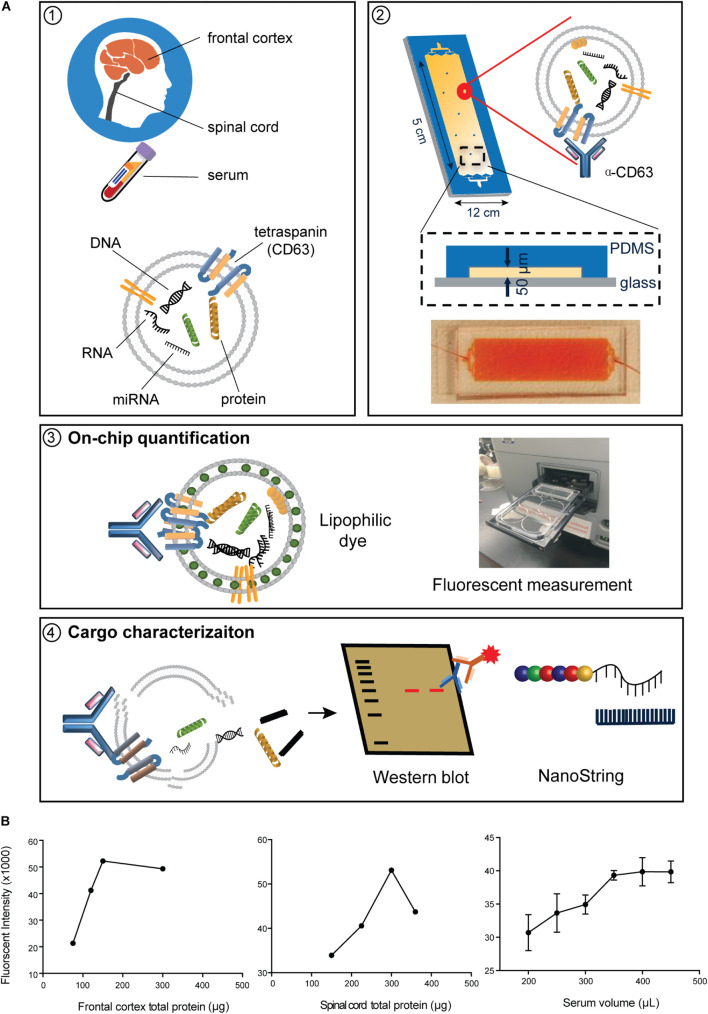
Immuno-affinity-based microfluidic EV isolation from ALS tissue. **(A)** Workflow for immobilizing and characterizing EVs. (1) EVs were captured from frontal cortex, spinal cord, and serum from ALS or control participants by a (2) CD63-antibody-coated ExoChip. (3) To quantify the captured EVs, the chip was processed with lipophilic (DiO) staining, and measured fluorescence intensity was normalized to background and correlated to the amount of fluorescently stained EVs. (4) Immobilized EVs were lysed for Western blotting and cargo profiling by NanoString. **(B)** Verification of the saturation capacity of the ExoChip device for serum and tissue homogenates. For frontal cortex and spinal cord, testing of 300 μL of homogenates with various total protein concentrations revealed a saturation concentration of 0.5 μg/μL for frontal cortex and 1 μg/μL for spinal cord tissue. For serum, the device reaches maximum fluorescence intensity when the volume of serum equals 400 μL. Data are presented as mean ± standard error of the mean (s.e.m.) (*n* = 3).

Using our refined ExoChip coated with CD63 antibody, we captured EVs and their cargo from frozen postmortem frontal cortex and spinal cord and from serum from ALS and control participants (Figure 1A). Saturation experiments indicated that 150 μg of frontal cortex, 300 μg of spinal cord, and 400 μL of serum resulted in device saturation (Figure 1B). These results informed the subsequent EV capture paradigms used to determine EV numbers, which required unsaturated conditions, and for cargo determination, where we used saturated conditions (see below).

**FIGURE 2:**
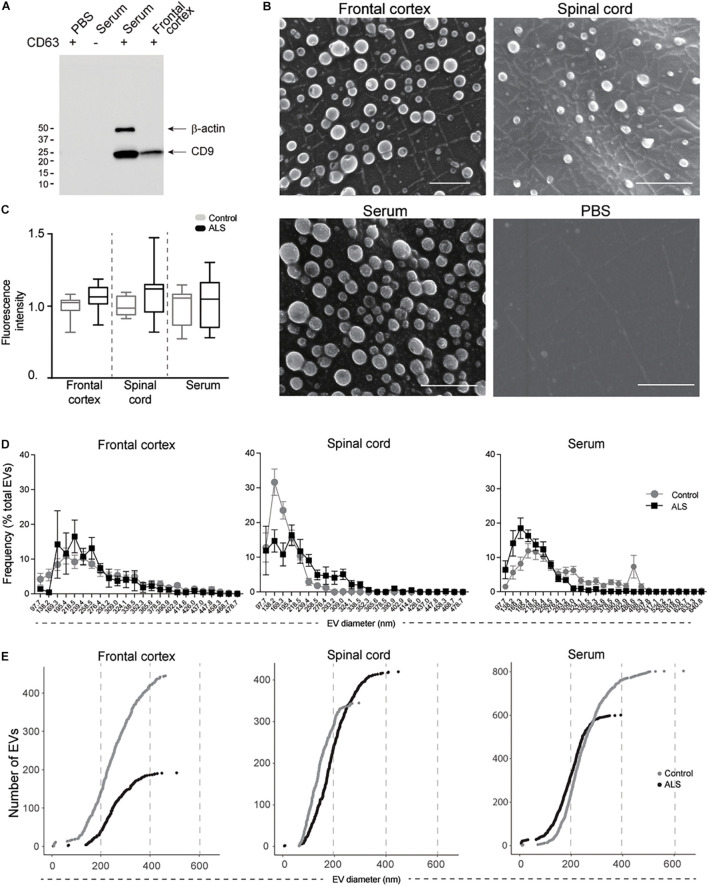
EV characterization from frontal cortex, spinal cord, and serum of ALS and control participants. EVs were captured by ExoChip. **(A)** Protein characterization from frontal cortex and serum EVs captured from anti-CD63 coated or non-coated devices. Immunoblotting was performed for β-actin and CD9. **(B)** SEM micrographs (bars = 1 μm). **(C)** On-chip immobilized and purified EVs were labeled with a fluorescent lipophilic dye (DiO). The fluorescence intensity values were normalized against the background. Fold-change is shown for minimum and maximum values with mean in a whisker plot. Frontal cortex (ALS = 12, Control = 8), spinal cord (ALS = 12, Control = 6), and serum (ALS = 7, Control = 9). **(D)** Size profile and frequency (%) by EV size measured from SEM micrographs in **(B)**. Data are presented as mean ± s.e.m.; ALS (black), healthy controls (gray). Frontal cortex (ALS = 191 EVs from 2 ALS participants, Control = 446 EVs from 3 healthy controls), spinal cord (ALS = 325 EVs from 3 ALS participants, Control = 441 EVs from 3 healthy controls), and serum (ALS = 601 EVs from 3 ALS participants, Control = 804 EVs from 3 healthy controls). **(E)** Cumulative plots for each tissue show the number of EVs and their corresponding diameter for ALS (black) and control (gray) participants.

Immunoblot analysis indicated that ExoChip-captured EVs were positive for the EV marker CD9 (Figure 2A). Assessment of ExoChip-captured EV size and morphology using SEM revealed spherical EVs from anti-CD63 coated ExoChips, but not from uncoated or PBS control ExoChips, suggesting specific vesicle capture (Figure 2B). The overall diameter of EVs captured from frontal cortex ranged from 5.64 to 447.71 nm for ALS and 7.97–480.15 nm for controls, while EV diameter from spinal cord ranged from 5.64 to 448.17 nm for ALS and 62.46–317.58 nm for controls, and EV diameter from serum ranged from 56.39 to 392.25 μM for ALS and 9.77–636.03 nm for controls. The mean diameter of EVs from frontal cortex was 255.07 ± 5.25 nm for ALS and 245.86 ± 4.45 nm for control; from spinal cord 193.92 ± 3.42 nm for ALS and 141.94 ± 2.59 nm for control; and from serum 187.12 ± 2.74 nm for ALS and 247.33 ± 2.96 nm for control. Size-frequency profiles, cumulative distribution plots, and 3 separate linear mixed effects models with random participant intercepts identified significantly larger EVs in spinal cord from ALS participants versus controls (*P* = 0.003). This trend was reversed for serum, in which the size of ALS EVs was smaller than controls (*P* = 0.02) (Figures 2D,E). We did not detect significant differences in EV size between ALS participants and controls in frontal cortex (*P* = 0.59). Extracellular vesicle number quantification by measuring membrane-associated lipids by fluorescence intensity revealed no significant difference in EV number between ALS and controls across all samples (Figure 2C).

### Extracellular Vesicle miRNA Cargo Is Dysregulated in Amyotrophic Lateral Sclerosis

**FIGURE 3:**
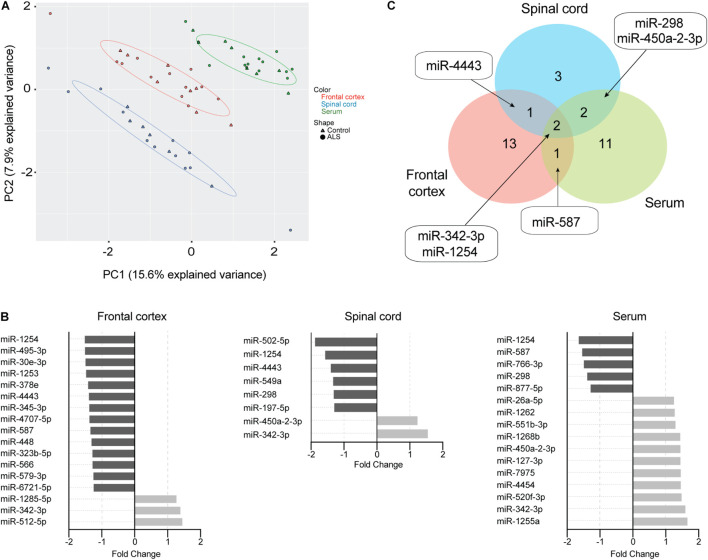
EV miRNAs are dysregulated in ALS frontal cortex, spinal cord, and serum. **(A)** Principal component analysis plot of unsupervised clustering of EV miRNA signal. **(B)** Fold-change of significant (*P* < 0.05) mature EV cargo DEmiRNAs was determined in ALS versus control groups. Increased fold-changes are represented in gray, while decreased fold-changes are represented in black. **(C)** Venn diagram showing common dysregulated EV-derived miRNAs from different ALS tissues. Frontal cortex (ALS = 12, Control = 8), spinal cord (ALS = 12, Control = 6), and serum (ALS = 14, Control = 8).

Mature miRNAs contained in ExoChip-captured EVs from frontal cortex, spinal cord, and serum were profiled by NanoString. Principal component analysis indicated that EV-derived miRNAs cluster according to tissue origin (Figure 3A). Using a geomean + 1SD cutoff as negative background and adjusting for batch effect, 33 significant DEmiRNAs (*P* < 0.05) were identified across the three tissues in ALS versus controls. We observed an overall decrease in EV-derived miRNA levels in ALS frontal cortex and spinal cord compared to controls, while the opposite was observed in serum (Figure 3B and Supplementary Table 2). In ALS frontal cortex, out of 17 dysregulated miRNAs, 3 increased and 14 decreased in level. In spinal cord, 8 miRNAs were dysregulated, with 2 increased and 6 decreased in ALS. In serum, 16 miRNAs were dysregulated, with 11 increased and 5 decreased in ALS. miRNA overlap across all three tissues (Figure 3C) revealed that miR-342-3p was increased and miR-1254 was decreased in ALS. Additionally, miR-4443 dysregulation overlapped between frontal cortex and spinal cord, miR-587 overlapped between frontal cortex and serum, and miR-298 and miR-450a-2-3p overlapped between spinal cord and serum.

### Biological Pathways Regulated by DEmiRNAs From Circulating or Neuronal Extracellular Vesicles in Amyotrophic Lateral Sclerosis

To better understand the role of dysregulated EV miRNAs in ALS pathology, we examined the functional KEGG pathways, which correspond to the predicted DEmiRNA targets for each tissue using DIANA-mirPath analysis (Vlachos et al., 2015). A total of 54 KEGG pathways were significantly enriched (FDR < 0.05) (Table 2). Of those pathways, 4 overlapped across all three tissues, and an additional 12 overlapped between two tissues, including axon guidance, long-term potentiation, transforming growth factor-beta (TGF-β)/mitogen-activated protein kinase (MAPK) signaling, and various cancer-associated pathways. The majority of the additional enriched pathways were in frontal cortex, where the hippo signaling pathway, Wnt signaling, and prion diseases were the most significantly enriched. Only 2 pathways were significantly enriched in spinal cord, and 4 pathways in serum.

**TABLE 2:**
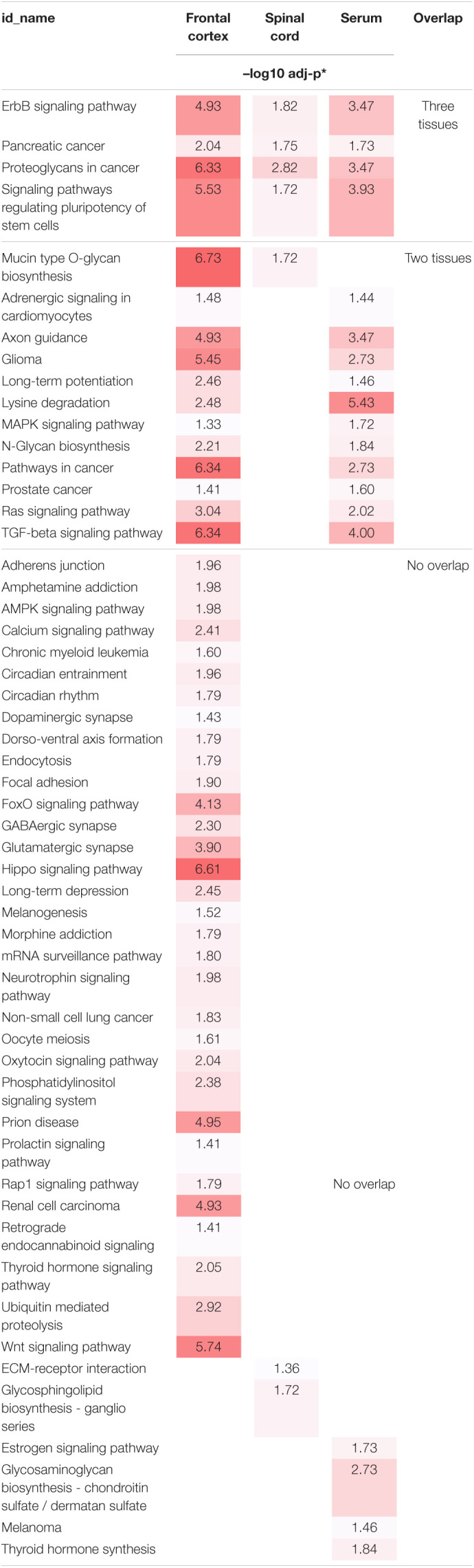
Individual and overlapping enriched KEEG Pathways of ALS DEmiRNA predicted targets from frontal cortex, spinal cord, and serum.

*Color-index represents significance levels ranging from no significance (white) to highest significance (dark red).

**FIGURE 4:**
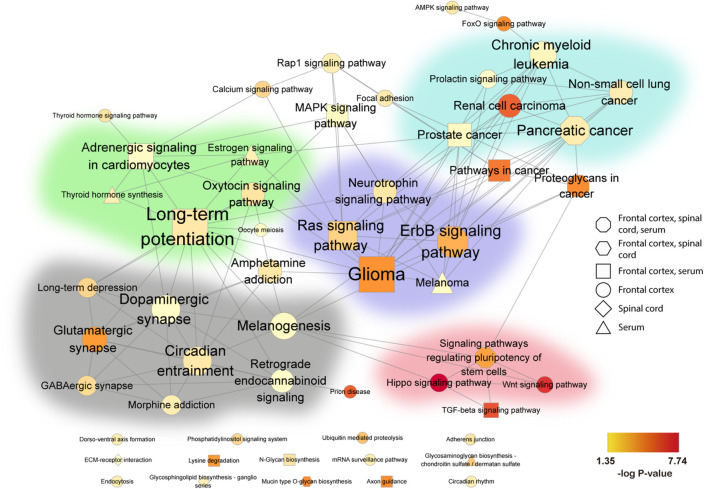
Association network of pathways in human ALS from frontal cortex, spinal cord, and serum. Significantly enriched KEGG pathways were combined and visualized in a network. KEGG pathways are represented by nodes, and shared gene content between pathways are represented by edges. Node shape indicates the tissue source of the enriched pathways: octagon (frontal cortex, spinal cord, serum), hexagon (frontal cortex, spinal cord), square (frontal cortex, serum), circle (frontal cortex), diamond (spinal cord), and triangle (serum). The size of each node denotes the number of miRNAs in that pathway, while the color gradient represents –log_10_(*P-*value) (see color scale, yellow to red). All networks were organized by the Edge-weighted Spring Embedded layout in Cytoscape with minimal manual node rearrangement for visibility. Background colors delimit highly inter-connected subnetworks identified by the Cytoscape MCODE app (Bader and Hogue, 2003): green (brain function), gray (neurotransmitter systems), purple (intracellular signaling cascades), blue (cancer related), and pink (stem cell renewal). Non-clustered pathways are arranged at the bottom. FC, frontal cortex; SC, spinal cord.

Connectedness across the identified pathways was then evaluated by the MCODE Cytoscape network analysis app. Connection between nodes (edges) denotes the degree of shared genes between two connected pathways. We identified five pathway clusters with highly interconnected common functional pathways (Figure 4): (1) neurotransmitter systems, such as “glutamatergic synapses” and “GABAergic synapses;” (2) brain function, including “long-term potentiation” and “oxytocin signaling;” (3) intracellular signaling cascades, such as “Ras signaling,” “neurotrophin signaling,” and “ErB signaling;” (4) cancer related, such as “prostate cancer,” “pancreatic cancer,” chronic myeloid leukemia;” and (5) stem cell renewal, including “hippo signaling,” TGF-beta signaling,” and “Wnt signaling.”

### Gene Targets and Biological Relevance of Dysregulated Extracellular Vesicle-Associated miRNAs in Amyotrophic Lateral Sclerosis

**FIGURE 5:**
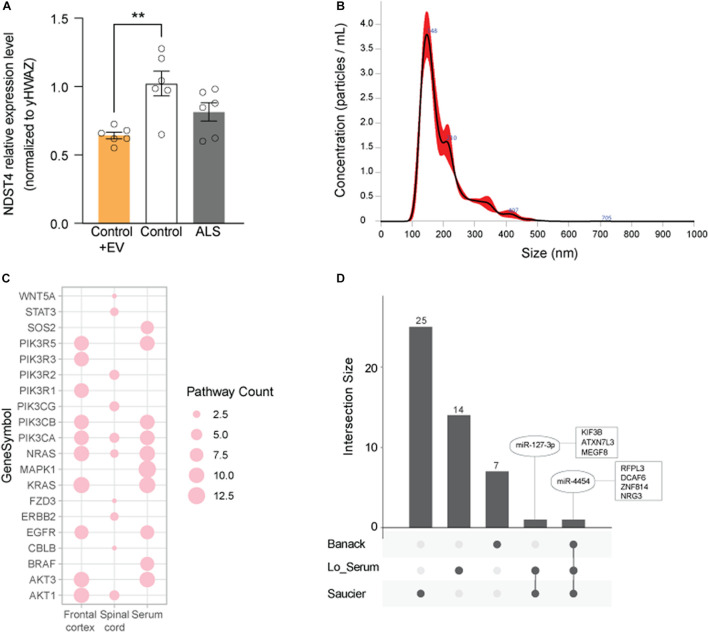
Evaluation of predicted gene targets associated with EV DEmiRNAs. **(A)** Top 10 genes represented among pathways associated with the DEmiRNAs from frontal cortex (FC), spinal cord (SC), and serum. Node size represents the number of associated pathways containing that gene target (see Supplementary Tables 3–5). **(B)** qPCR for NDST4 in ALS and control iNeurons (one cell line per condition), or in control iNeurons incubated with ALS spinal cord EVs. Results are normalized to an internal reference (YWHAZ) and presented as fold change calculated by the 2^−ΔΔCT^ method. Data represent mean ± standard error of the mean from 6 independent experiments. **P < *0.01* compared to the control group. **(C)** Size distribution of ALS spinal cord EV particles used in **(B)** determined by NanoSight. **(D)** EV DEmiRNAs overlapping across published studies (see Table 3), where common DEmiRNAs are indicated in ovals, and predicted or verified targets are indicated in squares. Dark gray circles correspond to EV DEmiRNAs unique to each data set; connections between circles correspond to DEmiRNAs overlapping across two or more data sets.

To assess potential biological relevance, we compared the predicted target mRNAs of the enriched KEGG pathways in each tissue (Figure 5A and Supplementary Tables 3–5). Transcripts among the most common predicted target genes from the enriched pathways were: serine-threonine kinases, including protein kinase C α/β (PRKCα/β), MAPK1, and AKT3; phosphoinositide 3-kinase (PI3K) subunits, including PIK3CB and PIK3R5; and Ras family GTPases, including NRAS and KRAS.

Since miRNAs regulate mRNA steady-state levels by destabilizing transcripts, we next assessed expression of NDST4, a predicted target of miR-520f-3p (increased in ALS serum EVs, Figure 3B), in an *in vitro* ALS model. Quantitative real-time PCR of ALS and control participant-derived iNeurons (Weskamp et al., 2020) confirmed that NDST4 gene expression trends to decrease 0.80-fold in ALS versus control iNeurons. This was similar to reduced NDST4 levels in postmortem ALS spinal cord tissue in a previous study (Figueroa-Romero et al., 2012). Additionally, incubating control iNeurons with ALS spinal cord EVs, size verified by NanoSight (Figure 5C), significantly reduced NDST4 expression 0.63-fold (Figure 5B) versus control iNeurons not incubated with ALS EVs.

## Discussion

EVs are secreted by all cell types and transport diverse cargos between cells and tissues. While critical for normal biological functions, EVs also participate in and promote disease states. In this study, we addressed the potential roles of EVs in ALS using ExoChip immno-affinity microfluidic technology to isolate EVs from serum and post-mortem CNS tissues from ALS cases and controls. Our results verify that this strategy provides an effective isolation platform to evaluate EV characteristics and compare EV miRNA cargo for human tissues and biofluids. Specifically, we observed differences in EV size between ALS and control participants in both circulating and CNS EVs. We also identified a subset of DEmiRNAs in EVs that overlap between serum and CNS tissue, and uncovered signaling pathways associated with the predicted targets of the identified DEmiRNAs across tissues. As increasing evidence supports the contention that dysregulated miRNAs exert important functions in disease pathology (Paez-Colasante et al., 2015; Wang and Zhang, 2020; Akbari Dilmaghani et al., 2021), these results suggest a role of miRNA cargo from circulating EVs in ALS pathogenesis and also highlight the potential ability of EVs to serve as ALS biomarkers.

EV isolation from human frontal cortex, spinal cord, and serum samples from our cohort of ALS and control participants demonstrated that numbers of EVs did not significantly differ between ALS and control samples, regardless of tissue type. This agrees with findings of comparable EV numbers in plasma and CSF from sporadic ALS versus healthy control participants (Sproviero et al., 2018; Thompson et al., 2020), and with a report of similar EV numbers from brain and spinal cord tissue of SOD1^G93A^ ALS mice versus non-transgenic littermates (Silverman et al., 2019). To our knowledge, EV numbers had not yet been examined in CNS tissue from ALS participants. We detected significant differences in EV size in spinal cord and serum between ALS cases and controls. Specifically, we found larger EVs in spinal cord of ALS participants when compared to controls, and smaller EVs in serum of ALS participants. These serum results are in the opposite direction of those obtained using nanoparticle-particle tracking analysis to quantitate EVs in ALS versus control plasma (Sproviero et al., 2018). The differences between studies, especially among control participants, could stem from the differing isolation and size determination methodologies as well as biological factors, including age, sex, tissue origin, cargo, time of sample collection, EV phenotype, and disease state. For example, detected EV size differences between serum and CNS tissues may relate to the fact that the tissue samples are all postmortem and collected at end-stage disease, whereas serum samples are obtained throughout the ALS disease course. It is also possible that EV size could impact cargo or vice versa. One report demonstrated that cancer-associated gene and protein levels were increased in small EVs relative to large EVs (Jeong et al., 2019), and we found that ALS spinal cord EVs, which were larger than control EVs, contained mostly downregulated miRNAs whereas ALS serum EVs, which were smaller than control EVs, were enriched in upregulated miRNAs. Ultimately, it remains unknown what the biological consequences are of EV size heterogeneity (Margolis and Sadovsky, 2019).

Evaluation of EV miRNA cargo revealed 33 miRNAs that were dysregulated in frontal cortex, spinal cord, and/or serum in ALS cases versus controls. The majority in ALS CNS tissues were reduced, while most in serum were elevated. Notably, miR-342-3p was increased in all three ALS tissues. In line with these findings, miR-342-3p levels are increased in Alzheimer’s disease patient hippocampal samples, and miR-342-3p suppression improves outcomes in Alzheimer’s disease and arkinson’s disease mouse models (Fu et al., 2019; Wu et al., 2019). miR-342-3p in serum EVs likewise has diagnostic value in multiple sclerosis (Ebrahimkhani et al., 2017), further supporting the potential relevance of this miRNA to neurodegeneration. Intriguingly, miR-342-3p is associated with prion-based neurodegeneration (Saba et al., 2008; Montag et al., 2009) and is present in EVs released from prion-infected neurons (Bellingham et al., 2012). This is in line with the previously mentioned contention that prion-like transfer of pathogenic cargos, including misfolded and aggregated translation products of mutated SOD1, TAR DNA-binding protein of 43 kDa (TDP-43), fused in sarcoma, or *C9orf72*, may have important implications for propagating neurodegeneration in ALS (Soria et al., 2017; McAlary et al., 2020).

miR-1254 was also altered across all three tissues in our study, exhibiting decreased levels in ALS frontal cortex, spinal cord, and serum. To our knowledge, this is the first time miR-1254 has been linked to the nervous system. However, studies have tied miR-1254 to myocardial infarction and heart failure (Bayes-Genis et al., 2018; de Gonzalo-Calvo et al., 2018; van Boven et al., 2018), as well as certain cancers (He et al., 2019; Jiang et al., 2019) where it is linked to Wnt signaling. Interestingly, recent evidence has tied the Wnt pathway to CNS-related functions, processes, and disorders, including ALS (Gonzalez-Fernandez et al., 2020). Thus, additional studies on how miR-1254 and its downstream targets potentially impact neuronal health in ALS may be warranted.

Additional miRNAs highlighted in our study that could reflect pathogenic events and serve as potential ALS biomarkers are miR-450a-2-3p, miR-587, and miR-298, as their changes in the CNS were paralleled in serum. miR-450a-2-3p and miR-587 have limited information available in the context of the CNS, but both have reported links to the TGF-β/MAPK regulatory pathway (Jahangirimoez et al., 2020; Liu et al., 2020), in line with our pathway analysis. TGF-β is a cytokine with multifaceted effects known to be dysregulated in neurodegenerative disorders, and influences motor neuron survival, susceptibility to glutamate excitoxicity, and non-cell autonomous mechanisms in ALS (Peters et al., 2017; Meroni et al., 2019; Galbiati et al., 2020; Russo and Wharton, 2021). miR-298 is perhaps the most studied of the overlapping DEmiRNAs and is linked to a range of neurologic conditions, including nerve crush injury, spinal bulbar muscular atrophy, and multiple sclerosis (Motti et al., 2017; Pourshafie et al., 2018; Gnanakkumaar et al., 2019). Additionally, miR-298 is known or predicted to target key proteins associated with Alzheimer’s disease, and its levels are reduced in Alzheimer’s disease postmortem temporal lobe tissue (Boissonneault et al., 2009; Chopra et al., 2020). miR-298 exhibits further biological relevance through its association with sirtuin-3 (SIRT3) (Li et al., 2019), which impacts mitophagy, reactive oxygen species, and toxic protein accumulation (Zhang et al., 2020), known mechanisms involved in ALS, Alzheimer’s disease, and Parkinson’s disease pathogenesis. Collectively, while miRNAs with both novel and established links to neurological disease and ALS were uncovered, the concordance of these DEmiRNA changes between CNS and serum EVs in sporadic ALS highlights the potential of EV-cargo miRNAs to offer pathogenic insight and serve as potential biomarkers.

Biological relevance can also be gleaned from the pathways and targets linked to DEmiRNAs from neuronal and circulating ALS EVs. In our pathway analysis, we observed multiple associated biological processes that primarily fell into five highly interconnected clusters. Pathways in cancer were common across the various samples, as were pathways related to neurologic function. Of particular interest, the ErbB signaling pathway overlapped between all three tissues. This pathway regulates cell proliferation, migration, differentiation, and apoptosis via MAPK, Akt, and related pathways. ErbB4 is also implicated in familial and sporadic ALS and altered in sporadic ALS spinal cord tissue (Takahashi et al., 2013, 2019; Dols-Icardo et al., 2018; Narain et al., 2019). ErbB tyrosine kinase receptors bind neuregulins, neurotrophic factors highly expressed in the CNS, which play a critical role in motor neuron function and survival (Mautino et al., 2004; Schram et al., 2020). A range of studies have targeted the ErbB/neuregulin axis as a therapeutic strategy for ALS (Liu et al., 2018; Modol-Caballero et al., 2020a, b; Tavassoly et al., 2020). Notably, ErbB4 is a target of miR-342, one of the DEmiRNAs identified across all three assessed tissues. NRG3 is a target of miR-4454, a DEmiRNA identified in the current study, as well as in two previous reports (Saucier et al., 2019; Banack et al., 2020).

Other pathways and targets central to ALS pathogenesis and neurodegeneration were additionally identified herein. The TGF-β pathway, discussed above, and pathways associated with neurotransmitter systems (*e.g*., glutamatergic and GABAergic synapses) are in line with the altered inhibitory/excitatory balance present in ALS (Schutz, 2005; Ramirez-Jarquin and Tapia, 2018; Gunes et al., 2020). Identification of axon guidance pathways reflect essential roles in neurocircuitry and synaptic connection development, processes dysregulated in ALS (Korner et al., 2019). Metabolic dysfunction pathways (*e.g*., mTOR signaling) and prion disease pathways are also consistent with known mechanisms in ALS participants (Saxton and Sabatini, 2017; Soria et al., 2017; McAlary et al., 2020). Likewise, Ras, PI3Ks, Akt, and PRKCs all have established roles in neuronal function and survival pathways of potential relevance to ALS (Recabarren-Leiva and Alarcon, 2018; Lanuza et al., 2019; Qu et al., 2019). We further confirmed that NDST4, a serum DEmiRNA target which is reduced in postmortem ALS spinal cord tissue (Figueroa-Romero et al., 2012), decreased in iNeurons treated with EVs isolated from sporadic ALS participant postmortem spinal cord. These data verify that sporadic ALS spinal cord-derived EVs can alter expression of a gene target previously implicated in ALS, attesting to potential biological relevance.

Finally, direct comparison of our complete dataset to other ALS EV-associated DEmiRNA profiles in the literature identified two overlapping miRNAs (Table 3 and Figure 5D). However, directionality of dysregulation in both cases was inconsistent. For miR-4454, we observed elevated levels in ALS serum while Banack, et al. and Saucier, et al. reported reduced levels in ALS plasma and serum EVs (Saucier et al., 2019; Banack et al., 2020). Of relevance to ALS, predicted miR-4454 targets have important associations with neurogenesis, synapse formation, and motor neuron integrity (Chen et al., 2018; Muller et al., 2018). Intriguingly, miR-4454 dysregulation has also been recently tied to particulate matter (PM_2_._5_) exposure (Mancini et al., 2020), of relevance to the increasing interest in the role of environmental exposures on ALS risk (Goutman and Feldman, 2020; Nunez et al., 2021). miR-127-3p was similarly elevated in our ALS serum samples but reduced in plasma samples from the Saucier et al. study (Saucier et al., 2019). This miRNA is part of a diagnostic exosomal biomarker panel for multiple sclerosis (Ebrahimkhani et al., 2017), and is further implicated in neurodegeneration through effects on neurite outgrowth, nerve injury, and apoptosis (He et al., 2016). miR-127-3p targets have known effects on axon guidance, histone acetylation, and intracellular motor proteins, as well as links to ALS (Engelhard et al., 2013; Andres-Benito et al., 2017; Nicolas et al., 2018). Collectively, our results indicate that circulating and CNS EVs from ALS participants contain DEmiRNAs with potential biological relevance to disease pathogenesis and could be useful biomarkers.

**TABLE 3:**
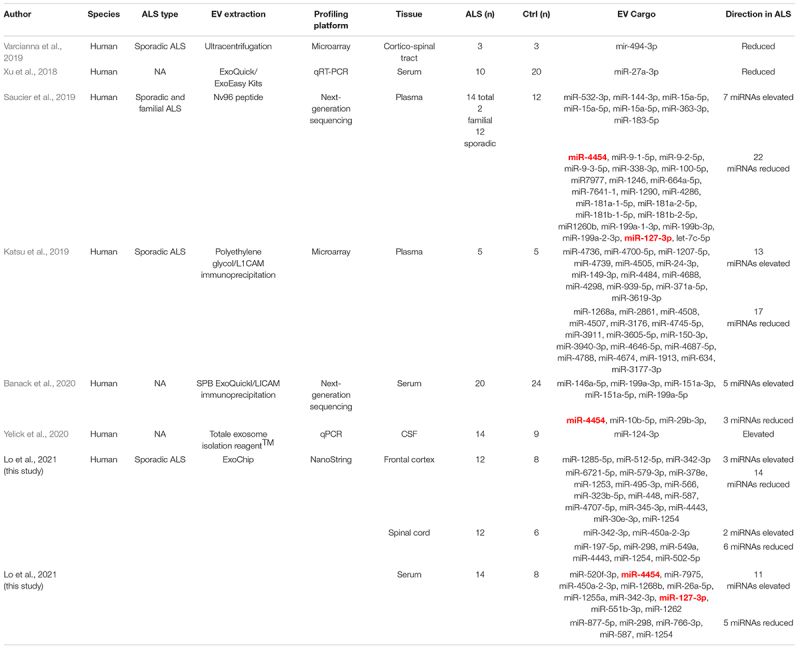
Studies reporting dysregulated EV miRNAs in ALS. Extracellular vesicle characterization in sporadic ALS

*Highlighted DEmiRNAs (bold/red) are shared amongst different studies.*

For the current study, we acknowledge that the sample size for each tissue was relatively small and limited to a heterogeneous population of ALS participants with sporadic disease. This advocates for future, larger studies in independent cohorts, which are powered to consider other disease determinants, such as disease progression rates, site of onset, family history, and genetic background. However, 11 of the ALS participants in our cohort provided all three tissues, and both frontal cortex and spinal cord were obtained from 4 of the control participants, which mitigated heterogeneity to a certain extent. We also recognize that comparing serum to postmortem tissues reflects samples obtained at different disease stages, as the blood was collected from living individuals. Longitudinal studies with serial sampling will help delineate whether the EV miRNA profiles change throughout the disease course. Additionally, while CD63 is a commonly accepted EV marker, a CD63-negative EV population has been reported (Lee et al., 2018). Thus, antibodies against neuronal-specific antigens, such as L1 cell adhesion molecule (L1CAM) and the GluR2/3 subunits, could be considered in future studies to capture and study EVs of neuronal origin (Zhang and Chopp, 2016; Banack et al., 2020).

Overall, we identified novel and established EV-associated miRNAs, pathways, and targets with potential importance in ALS, supporting the contention that EVs likely have a role in disease pathogenesis. This is evidenced by our discovery of associated pathways with known relevance to ALS, and validation of altered target expression in iNeurons exposed to ALS EVs that support biological relevance. Our data demonstrating that circulating EV miRNA cargo mirror those of the CNS disease state in ALS further highlights the potential of EV miRNAs as prognostic and diagnostic biomarkers. Thus, future studies focused on the current DEmiRNAs as well as efforts to identify and construct a unique miRNA panel capable of distinguishing ALS from other neurodegenerative diseases are warranted. Moreover, the ability of EVs to carry molecular information from one cell to another provides an opportunity to leverage proteomic and genetic information for neurodegenerative disease pathogenic insights and diagnostics (Chen et al., 2019; Hosaka et al., 2019; Meldolesi, 2021).

## Data Availability Statement

The datasets presented in this study can be found in online repositories. The names of the repository/repositories and accession number(s) can be found below: https://www.ncbi.nlm.nih.gov/geo/, GSE179819.

## Ethics Statement

The studies involving human participants were reviewed and approved by University of Michigan Medical School Institutional Review Board. The patients/participants provided their written informed consent to participate in this study.

## Author Contributions

CF-R, SG, SN, and EF: conception and design of the study. T-wL, CF-R, CP, JH, ES, CS, RL, AN, and SG: acquisition and analysis of data. CF-R, T-wL, SS, and EF: drafting a significant portion of the manuscript or figures. All authors contributed to the article and approved the submitted version.

## Conflict of Interest

SG sat on an advisory board for Biogen and IFT Pharma and serves on a Data Safety Monitoring Board. The remaining authors declare that the research was conducted in the absence of any commercial or financial relationships that could be construed as a potential conflict of interest.

## Publisher’s Note

All claims expressed in this article are solely those of the authors and do not necessarily represent those of their affiliated organizations, or those of the publisher, the editors and the reviewers. Any product that may be evaluated in this article, or claim that may be made by its manufacturer, is not guaranteed or endorsed by the publisher.

## Footnotes

1
https://github.com/hurlab/richR

2
https://www.R-project.org/
