# Supplementary material for: Extracellular Vesicles in Serum and Central Nervous System Tissues Contain microRNA Signatures in Sporadic Amyotrophic Lateral Sclerosis
Source: Front Mol Neurosci. 2021 Oct 29;14:739016. doi: 10.3389/fnmol.2021.739016 (PMC8586523; doi:10.3389/fnmol.2021.739016)
Supplement: Supplementary file 1 [file Table_1.DOCX]

| **Supplementary Table S1. Individual clinical characteristics of study participants** | | | | | | | | |
| --- | --- | --- | --- | --- | --- | --- | --- | --- |
| **Sample#** | **Tissue** | **Condition** | **Sex** | **Ethnicity** | **Age at death** | **Onset** | **PMI (hrs)** | **SC segment** |
| 1 | S | ALS | F | C | 47 | Lumbar | NA |  |
| 2 | S | ALS | M | C | 78 | Bulbar | NA |  |
| 3 | S | ALS | M | C | 71 | Cervical | NA |  |
| 4 | SC | ALS | F | C | 73 | Lumbar | 24 | Cervical |
| 5 | SC/FC/S | ALS | F | C | 57 | Lumbar | 17 | Cervical |
| 6 | SC/FC/S | ALS | F | C | 65 | Lumbar | 9 | Cervical |
| 7 | SC/FC/S | ALS | F | C | 61 | Lumbar | 8 | Thoracic |
| 8 | SC/FC/S | ALS | F | C | 80 | Lumbar | 11 | Cervical |
| 9 | SC/FC/S | ALS | F | C | 68 | Lumbar | 23 | Cervical |
| 10 | SC/FC/S | ALS | M | C | 76 | Cervical | 9 | Cervical |
| 11 | SC/FC/S | ALS | M | C | 54 | Lumbar | 14 | Cervical |
| 12 | SC/FC/S | ALS | M | C | 63 | Cervical | 9 | Cervical |
| 13 | SC/FC/S | ALS | M | C | 68 | Cervical | 4 | Cervical |
| 14 | SC/FC/S | PLS | M | C | 54 | Lumbar | NA | Cervical |
| 15 | SC/FC/S | ALS | M | C | 51 | Lumbar | 23 | Cervical |
| 16 | FC | Cntl | F | C | 69 |  | 16 |  |
| 17 | FC | Cntl | F | C | 53 |  | 11 |  |
| 18 | FC | Cntl | M | C | 59 |  | 12 |  |
| 19 | S | Cntl | F | C | 50 |  | NA |  |
| 20 | S | Cntl | F | C | 80 |  | NA |  |
| 21 | S | Cntl | F | C | 68 |  | NA |  |
| 22 | S | Cntl | M | C | 49 |  | NA |  |
| 23 | S | Cntl | M | C | 72 |  | NA |  |
| 24 | S | Cntl | M | C | 56 |  | NA |  |
| 25 | S | Cntl | M | C | 60 |  | NA |  |
| 26 | SC | Cntl | M | C | 55 |  | 20 |  |
| 27 | SC | Cntl | M | C | 65 |  | 14 |  |
| 28 | SC/FC | Cntl | F | AA | 80 |  | 26 |  |
| 29 | SC/FC | Cntl | F | C | 85 |  | 21 |  |
| 30 | SC/FC | Cntl | M | C | 82 |  | 6 |  |
| 31 | SC/FC | Cntl | M | C | 70 |  | 21 |  |
| FC, frontal cortex; SC, spinal cord; S, serum, Cntl, control; F, female; M, male; C, Caucasian; AA, African American; PMI, postmortem interval; hrs, hours; NA, data not available | | | | | | | | |
