# Supplementary material for: Extracellular Vesicles in Serum and Central Nervous System Tissues Contain microRNA Signatures in Sporadic Amyotrophic Lateral Sclerosis
Source: Front Mol Neurosci. 2021 Oct 29;14:739016. doi: 10.3389/fnmol.2021.739016 (PMC8586523; doi:10.3389/fnmol.2021.739016)
Supplement: Supplementary file 2 [file Table_2.DOCX]

| **Supplementary Table S2. DEmiRNAs from ALS frontal cortex, spinal cord, and serum** | | | |
| --- | --- | --- | --- |
| **DEmiRNA** | **Tissue** | **Fold change** | ***P*-value** |
| miR-512-5p | FC | 1.45 | 0.044 |
| miR-342-3p | FC | 1.39 | 0.032 |
| miR-1285-5p | FC | 1.27 | 0.027 |
| miR-6721-5p | FC | -1.24 | 0.044 |
| miR-579-3p | FC | -1.25 | 0.026 |
| miR-566 | FC | -1.28 | 0.024 |
| miR-323b-5p | FC | -1.28 | 0.038 |
| miR-448 | FC | -1.31 | 0.046 |
| miR-587 | FC | -1.34 | 0.038 |
| miR-4707-5p | FC | -1.37 | 0.007 |
| miR-345-3p | FC | -1.38 | 0.020 |
| miR-4443 | FC | -1.39 | 0.009 |
| miR-378e | FC | -1.41 | 0.003 |
| miR-1253 | FC | -1.47 | 0.023 |
| miR-30e-3p | FC | -1.49 | 0.017 |
| miR-495-3p | FC | -1.51 | 0.002 |
| miR-1254 | FC | -1.51 | 0.013 |
| miR-342-3p | SC | 1.55 | 0.003 |
| miR-450a-2-3p | SC | 1.23 | 0.043 |
| miR-197-5p | SC | -1.29 | 0.010 |
| miR-298 | SC | -1.31 | 0.047 |
| miR-549a | SC | -1.33 | 0.003 |
| miR-4443 | SC | -1.40 | 0.025 |
| miR-1254 | SC | -1.57 | 0.012 |
| miR-502-5p | SC | -1.88 | 0.000 |
| miR-1255a | S | 1.66 | 0.014 |
| miR-342-3p | S | 1.60 | 0.003 |
| miR-520f-3p | S | 1.48 | 0.038 |
| miR-4454 | S | 1.46 | 0.040 |
| miR-7975 | S | 1.46 | 0.040 |
| miR-127-3p | S | 1.45 | 0.040 |
| miR-450a-2-3p | S | 1.45 | 0.010 |
| miR-1268b | S | 1.44 | 0.034 |
| miR-551b-3p | S | 1.30 | 0.050 |
| miR-1262 | S | 1.27 | 0.046 |
| miR-26a-5p | S | 1.25 | 0.039 |
| miR-877-5p | S | -1.28 | 0.027 |
| miR-298 | S | -1.39 | 0.004 |
| miR-766-3p | S | -1.49 | 0.006 |
| miR-587 | S | -1.54 | 0.002 |
| miR-1254 | S | -1.64 | 0.002 |
